# Genetic Evolution of Antibiotic Resistance and Virulence Genes in *Escherichia coli* Isolates from a Chinese Hospital over a 12-Year Period

**DOI:** 10.3390/microorganisms13040954

**Published:** 2025-04-21

**Authors:** Chengjie Feng, Hongbing Jia, Qian Yang, Qinghua Zou

**Affiliations:** 1 Department of Microbiology, School of Basic Medical Sciences, Peking University, Beijing 100191, China; 2 Department of Clinical Laboratory, China-Japan Friendship Hospital, Beijing 100029, China; 3 Chinese Center for Disease Control and Prevention, Beijing 102206, China

**Keywords:** *Escherichia coli*, antibiotic resistance, virulence, genetic evolution

## Abstract

*Escherichia coli* is a significant pathogen capable of inducing a variety of infections in both human and animal hosts. Despite its clinical significance, there is a lack of longitudinal research aimed at elucidating the genomic attributes that facilitate antimicrobial resistance and clonal dissemination in this bacterium. In this study, we investigated the genetic dynamics of antibiotic resistance and virulence factors within a collection of 137 *E. coli* isolates retrieved from a Chinese hospital over a 12-year period. Notably, a substantial increase in resistance to various antibiotics, including broad-spectrum β-lactams, aminoglycosides, and quinolones, was observed. Additionally, our study revealed the acquisition of diverse antibiotic resistance and virulence genes across different sequence types (STs). Among the STs, ST131 emerged as the most prevalent, exhibiting a high level of multidrug resistance. In contrast, ST73 and ST12 demonstrated a higher prevalence of virulence genes, suggestive of a potential trade-off between antibiotic resistance and virulence. What’s more, we identified significant intra-clonal diversification and convergence of antibiotic resistance and virulence traits within the dominant ST131 group. These findings underscore the importance of longitudinal studies in understanding the evolution of bacterial pathogens and the necessity for ongoing research to inform public health strategies.

## 1. Introduction

*Escherichia coli*, a ubiquitous and versatile bacterial species, presents a formidable challenge to global public health due to its escalating levels of antibiotic resistance and virulence. Nosocomial infections caused by *E. coli* can range from urinary tract infections to bloodstream infections and neonatal meningitis infections. Despite the ongoing efforts to constrain the spread of this bacterium, *E. coli* has consistently ranked as the most common Gram-negative bacterium in community-acquired and nosocomial bacteremia [1,2]. The incidence of *E. coli* bloodstream infections (BSI) was reported to have increased by 76% from 2011 to 2015 in the United Kingdom [3]. Similar to other countries, *E. coli* also was the most frequent bacterial species among clinical isolates in China, accounting for 18.1% of all isolates in 2023 [4].

The ability of *E. coli* to acquire antibiotic resistance has been exacerbated by the widespread and sometimes indiscriminate use of antibiotics in clinical settings. Consequently, the prevalence of antibiotic-resistant *E. coli* has risen steadily [5,6], posing significant challenges to effective anti-infective therapy [7,8,9]. The acquisition of antibiotic resistance in *E. coli* is facilitated by various mechanisms, with gene mutation and horizontal gene transfer being pivotal pathways [10,11]. Notably, recent research suggests that gene acquisition, rather than mutation, plays a primary role in driving evolutionary changes within the gut environment [11]. Understanding the dynamics of horizontal gene transfer among different *E. coli* populations, along with the carriage of antibiotic elements, is crucial for unraveling the evolutionary patterns that govern *E. coli* antibiotic resistance.

In addition to its ability to develop antibiotic resistance, *E. coli* also exhibits significant virulence potential through the production of an array of toxins, including enterotoxins, hemolysins, and cytotoxins [12,13]. Virulence factors in *E. coli* related to urinary tract infection [14,15], bloodstream infection [16,17], and neonatal meningitis [18,19] have been widely studied. The continual evolution of virulence and antibiotic resistance genes in *E. coli* isolates has contributed to the increasing severity and incidence of infections [20,21].

Previous studies showed that the population structure of *E. coli* is predominantly clonal, allowing the delineation of major phylogenetic groups [20,21] such as ST131, ST73, ST95, and ST69. ST131 has received particular attention, following its apparent emergence in the 2000s, due to its rapid global dissemination and frequent multidrug resistance phenotype [22,23,24]. Some studies analyzed the virulence genes in *E. coli* isolates from neonates [25]; another analyzed the resistance patterns of *E. coli* according to patient age and clinical sample type [26]. Huang et al. compared the etiological profiles and antibiotic resistance patterns of UTIs sorted by different age categories from a tertiary general hospital during a 12-year period [27]. These studies provide important information for us to learn about the evolution of *E. coli.* However, they mainly concentrated on some specific genes, and large gaps remain in our understanding of the genomic features that contribute to its antimicrobial resistance and clonal dissemination. Longitudinal studies examining *E. coli* isolates over extended periods have been notably sparse. This study investigated the genetic evolution of antibiotic resistance and virulence genes in 137 strains of *E. coli* isolates at the whole genome level in a Chinese hospital from 2004 to 2015, to shed light on the intricate genetic mechanisms underlying these isolates.

## 2. Materials and Methods

### 2.1. Clinical Isolates

Clinical specimens (10 sputum, 11 urine, 66 blood, 11 ascites, 6 drain, 5 bilious, 2 pleural-effusion, 12 secretion, 14 other) were collected aseptically from hospitalized patients from 2004–2015, and were cultured on MacConkey agar (Oxoid, Hampshire, UK) at 37 °C for 24 h. Single colonies were assigned unique numbers and subcultured on blood agar (bioMérieux, Marcy-l’Étoile, France) for purity. Identification was confirmed using VITEK2 Compact (bioMérieux (Marcy-l’Étoile, France)) and MALDI-TOF MS (Bruker Daltonics, Billerica, MA, USA) with a score threshold of ≥2.0.

### 2.2. Antibiotic Susceptibilities

For antibiotic susceptibility testing, colonies were suspended in 0.45% saline to a 0.5 McFarland standard. Suspensions were loaded into VITEK2 GN cards (bioMérieux, Marcy-l’Étoile, France) and incubated at 35 °C for 8–12 h. Results were interpreted per CLSI 2023 guidelines. A total of ten major categories and 18 types of antibiotic agents were selected based on WHO AWaRe classification, local prescription patterns, and CLSI guidelines to represent major therapeutic classes, and antibiotic susceptibility was tested based on the interim standard definitions for acquired resistance [28], with the isolates classified into multidrug resistant (MDR) and sensitive isolates. MDR was defined as acquired non-susceptibility to at least one agent in three or more antimicrobial categories; isolates with non-susceptibility to only one or two categories were defined as sensitive isolates.

### 2.3. Whole-Genome Sequencing

Genomic DNA was extracted using the QIAamp DNA Mini Kit (Qiagen, Hilden, Germany). DNA quality was assessed via Nanodrop (A260/A280 ≥ 1.8) and Qubit (Thermo Fisher, Waltham, MA, USA). DNA libraries were prepared using the Nextera XT Kit (Illumina, San Diego, CA, USA). Fragmentation involved tagmentation at 55 °C for 5 min, followed by limited-cycle PCR (12 cycles) for index adapter ligation. Whole-genome sequencing was performed using the Illumina platform (Illumina Inc. (San Diego, CA, USA) by Shanghai Majorbio Pharmaceutical Technology Co., Ltd., Shanghai, China). Fragment libraries were constructed with an insertion size of approximately 400 bp and subjected to PE150 sequencing (paired-end sequencing with a read length of 150 bp) and 100× Illumina sequencing data, resulting in multiple genome scaffolds.

The original data were subjected to quality assessment using FastqTotalHighQualityBase.jar, SeqPrep, and Sickle software (version 1.33) to trim low-quality reads. Trimmomatic software (v0.36) (http://www.usadellab.org/cms/?page=trimmomatic (accessed on 1 January 2024)) was used to remove adapter sequence. High-quality reads obtained after quality trimming were referred to as Clean data, which were used for genome assembly and stored in fastq format.

SOAPdenovo2 software (https://sourceforge.net/projects/soapdenovo2/report_inappropriate (accessed on 1 January 2024)) were used to concatenate second-generation sequences (parameters: kmer: 21–47) to obtain contigs assembly results. The reads were then mapped to the contigs and finally formed into scaffolds. Coding sequences (CDS) were predicted using Glimmer (version 3.02), GeneMarkS (version 4.28), and Prodigal software (version 2.6.3). Plasmid identification and annotation were carried out using PlasFlow (version 1.1), BLAST v2.2.25, and PLSDB database (version 2024_05_31_2). BLAST+ was used for gene functional annotation based on selected reference genomes. The sequences were aligned and functionally annotated using Diamond, hmmer3, blast2go, and databases such as NR, Swiss-Prot, Pfam, EggNOG, GO, and KEGG.

### 2.4. Bioinformatics Analysis

IslandViewer (http://www.pathogenomics.sfu.ca/islandviewer/ (accessed on 1 January 2024)) was utilized to predict genomic islands, and Phage_Finder (https://phage-finder.sourceforge.net (accessed on 1 January 2024)) for prophages. Diamond software in combination with the Comprehensive Antibiotic Resistance Database (CARD) (http://arpcard.Mcmaster.ca (accessed on 1 January 2024), Version 1.1.3), Virulence Factors Database (VFDB) (http://www.mgc.ac.cn/VFs/main.htm (accessed on 1 January 2024)), and KEGG database were used for annotating analysis of antibiotic resistance genes, virulence genes, and secretion systems in each genome, respectively. Sequences with coverage > 80% and identity > 90% of virulence and antibiotic resistance genes were selected for further analysis.

PlasmidFinder-v2.0 and MLST (https://pubmlst.org (accessed on 1 January 2024)) databases were used for plasmid replicon typing and MLST typing identification through sequence alignment using BLAST, respectively. Pathogenwatch (https://pathogen.watch/ (accessed on 1 January 2024)) and BacWGSTdb 2.0 (http://bacdb.cn/BacWGSTdb/ (accessed on 1 January 2024)) were used for bacterial identification. Core genome SNPs were identified using Snippy v4.6.0 (parameters: --mincov 10, --minfrac 0.9) by aligning reads to the *E. coli* reference genome (NCBI: NC_000913.3). SNP alignments were analyzed in MEGA5 using the maximum likelihood method (GTR + G + I model, 1000 bootstrap replicates) to construct an evolutionary tree. The evolutionary tree was visualized using the online tool Chiplot (https://www.chiplot.online/ (accessed on 1 January 2024)). SNP differences, as depicted from the core genome alignment, were used for PCA analysis. The BLAST Ring Image Generator (BRIG) software (http://sourceforge.net/projects/brig (accessed on 1 January 2024)) was used to draw genome and plasmid circle diagrams based on BLAST+ (parameter: -evalue 1 × 10^−5^), and vector drawings were performed using EasyFig for analysis.

## 3. Results

### 3.1. Antibiotic Resistance of the E. coli Isolates

A total of 137 *E. coli* isolates from a teaching hospital in China from 2004 to 2015 were available in this study. The annual distribution, the sample source, and antibiotic resistance profiles of the isolates were detailed in Table S1. The number of isolates ranged from 8 to 15 each year, with a predominant presence of bloodstream isolates (67/137, 48.9%), followed by secretions (12/137, 8.7%). A total of ten major categories and 18 types of antibiotics were tested for antibiotic sensitivity. The antibiotic categories included aminoglycosides, antipseudomonal penicillins + β lactamase inhibitors, carbapenems, narrow-spectrum spectrum cephalosporins: first- and second-generation cephalosporins, extended-spectrum cephalosporins: third- and fourth-generation cephalosporins, cephamycins, fluoroquinolones, monobactams, penicillins, and penicillin + β-lactamase inhibitors. Notably, the isolates had high resistance to penicillins, aminoglycosides, fluoroquinolones, and non-extended spectrum cephalosporins, with a resistance rate of about 87%, 63%, 61%, and 54%, respectively, while the isolates exhibited a relative higher sensitivity to imipenem, cefotetan, amikacin, and piperacillin-tazobactam (Table S1), with sensitivities exceeding 90%. The majority of *E. coli* isolates (90 out of 137; 65.7%) showed acquired non-susceptibility to at least one agent in three or more antimicrobial categories and were classified as multidrug resistant (MDR) isolates, whereas 32 isolates (23.3%) showed relatively high sensitivity to all the antibiotics tested except one or two categories. The remaining 25 isolates were not classified due to their lack of antibiotic resistance profile.

### 3.2. Multilocus Sequence Types and Phylogenetic Relationships of the Isolates

**Figure 1:**
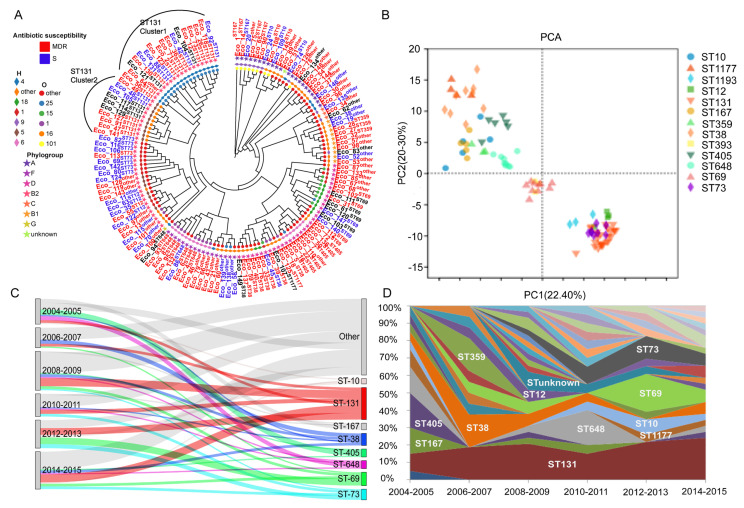
Phylogenetic analysis and the popularity trend of different sequence types over time. (**A**) Phylogenetic tree inferred using maximum likelihood for the 137 *E. coli* core genomes. The STs were marked with the superscript on the leave name, antibiotic sensitivity was indicated by the color of leave name, phylogroup and serogroup were marked with corresponding symbols; (**B**) PCA analysis of the 137 genomes. ST131, ST12, ST73, ST1193 (bottom right), ST393, ST69 (center), and Minor STs (e.g., ST359, ST167) (top left); (**C**,**D**) the popularity trend of different sequence types over time.

The 137 *E. coli* isolates were highly diverse; they were classified into 44 different sequence types, with ST131 being the most predominant (26/137, 18.9%), followed by ST69 (10/137, 7.3%), ST38 (9/137, 6.5%), ST648 (9/137, 6.5%), ST73 (8/137, 5.8%), ST405 (6/137, 4.4%), ST10 (5/137, 3.6%), and ST167 (5/137, 3.6%). O25:H4 was the predominant serotype (13%), followed by O16:H5 and O1:H6 (both 7%). O25:H4 and O16:H5 were primarily composed of ST131, while O1:H6 was mainly composed of ST648 and ST405. Phylogroup B2 accounted for the largest proportion (48, 35%), followed by D (35, 25.5%) and A (22, 16.1%). Phylogroup B2 was mainly composed of ST73 and ST131, and phylogroup D was mainly composed of ST38, ST405, and ST69 (Figure 1A, Table S1). The correlations of MDR vs. susceptible strains according to their classification into the phylogroups and STs are presented in Tables S2 and S3.

As shown by the core genome SNP phylogenetic tree, the 137 *E. coli* isolates were clustered into distinct lineages, which correlated with the phylogroups (Figure 1A). Most STs exhibited some degree of antibiotic resistance, with ST131, ST648, ST38, ST167, and ST405 being predominantly multidrug resistant. ST73 and ST12 were mainly sensitive isolates. Among these, ST131 were divided into two clusters: the first cluster (cluster 1: Eco_92, 118, 26, 73, 12, 104, 42, 36, 71, 2, 123, 88, 131, 130, 121, 56) and the second cluster (cluster 2: Eco_49, 48, 105, 117, 114, 129, 127, 91, 47, 141); ST69 was also split into two clusters. PCA analysis (Figure 1B) showed that all the isolates clustered into three main groups: ST131, ST12, ST73, and ST1193 comprised group 1, ST393 and ST69 comprised group 2, whereas the remaining STs were grouped separately, which is consistent with the corresponding ST clusters in the phylogenetic tree analysis. We then analyzed the popularity trend of different sequence types over time. ST131 has always been the mainstream sequence type, distributed in all time periods (Figure 1C). The proportion of ST38, ST405, and ST359 decreased year by year, as ST131, ST73, ST69, and ST10 increased (Figure 1D).

### 3.3. Antibiotic Resistance Genes in the Isolates

**Figure 2:**
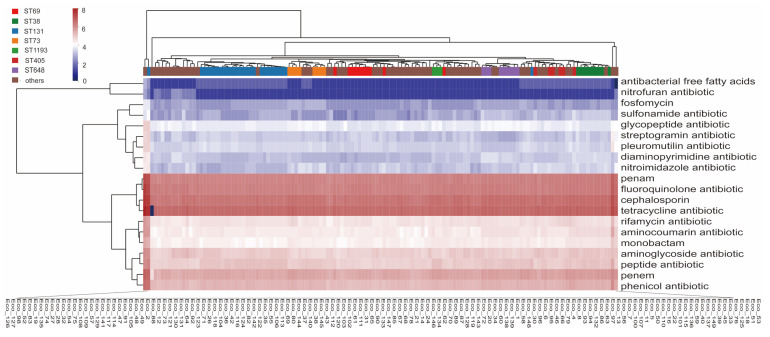
Distribution of antibiotic resistance genes related to different antimicrobial classes in different isolates. The antibacterial free fatty acids and nitrofuran antibiotic fosfomycin on the right refer to acquired genes conferring resistance to the antibiotic group. STs are marked at the top. Intensity of box shading indicates the number of antibiotic genes related to the classes of antimicrobials.

First, we compared the antibiotic resistance genes accounting for different categories of antibiotics such as antibacterial free fatty acids, nitrofuran antibiotic, fosfomycin, and sulfonamide. The results showed that the isolates have a large number of antibiotic resistance genes related to penems, fluoroquinolones, cephalosporins, and tetracyclines resistance, which is consistent with the clinical usage frequency of these antibiotics, and there were no significant differences in the number of these genes among different isolates. Disparities in resistance gene profiles emerged primarily within less commonly targeted antibiotic classes, including antibacterial free fatty acids, nitrofuran antibiotic, and fosfomycin (Figure 2).

### 3.4. Comparison of Antibiotic Resistance Genes Among Different STs

**Figure 3:**
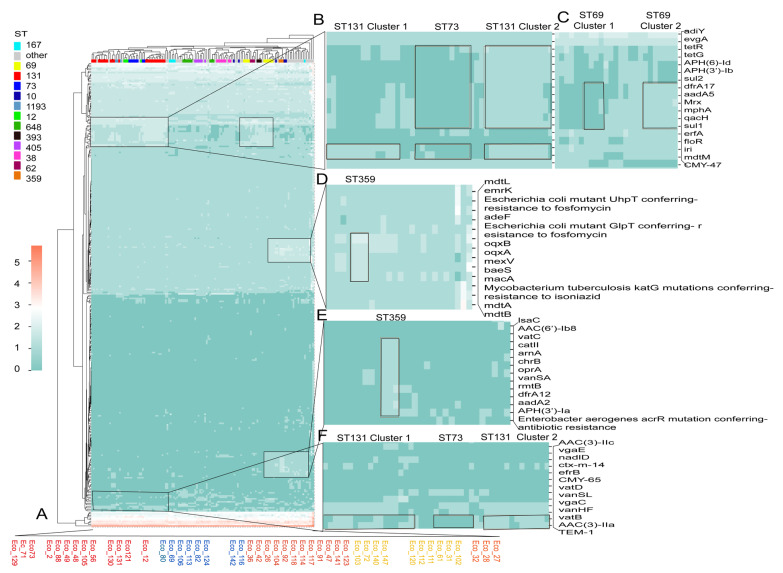
Clustering heatmap based on the Comprehensive Antibiotic Resistance Database (CARD) gene content. (**A**) Clustering heatmap, STs are marked at the top. Intensity of box shading indicates the number of antibiotic genes related to the CARD item; (**B**,**F**) the different CARD genes between ST131 cluster 1, cluster 2, and ST73; (**C**) the different CARD genes between the two clusters of ST69; (**D,E**) unique CARD gene items of ST359.

To look further into the difference in antibiotic resistance genes between different STs, we performed a clustering heatmap based on the Comprehensive Antibiotic Resistance Database (CARD) gene content. The results revealed that isolates of the same ST generally clustered together (Figure 3A), and different STs differ in CARD items. ST131 was predominantly divided into two clusters. The first cluster contained isolates of Eco_129, 71, 73, 2, 88, 49, 48, 105, 56, 130, 131, 121, and 12, and the second cluster contained isolates of Eco_36, 42, 26, 104, 92, 118, 114, 117, 91, 47, 141, and 123. The clustering of the isolates showed a marked difference with the clusters on the core genome tree, with Eco_129, 49, 48, 105, 36, 42, 26, 104, 92, 118, and 123 clustering differently from the core genome tree. The two clusters differ in the genes of *tetR/tetG/APH(6)-Id/APH(3″)-Ib/sul1/sul2*, which were present in cluster 2 but absent in cluster 1. ST73 is closer to cluster 1 than cluster 2 with the absence of these genes (Figure 3B). Both clusters of ST131, compared to ST73, are abundant in *iri* (monooxygenase, rifampicin resistance), *mdtM* (efflux pump, multiantibiotic resistance) (Figure 3B), *aac(3)-IIa,* and *bla*_TEM-1_ (Figure 3F). ST69 was also divided into two clusters. Cluster 1 lacks the resistant genes *dfrA17*, *aadA5*, *Mrx*, *mphA*, *qacH*, and *sul1* in comparison to cluster 2 (Figure 3C). All three ST359 isolates are MDR isolates. Correspondingly, Figure 3D,E demonstrated that ST359 possesses unique antibiotic resistance genes, including *oqxA/B*, *mexV*, *baeS*, *macA*, *vatC*, *catII*, *arnA*, *chrB*, *oprA*, *vanSA*, *rmtB*, *dfrA12*, *aadA2*, and *APH(3′)-Ia* genes, relative to other STs.

### 3.5. The Changing Pattern of Antibiotic-Resistant Gene Content over Time

**Figure 4:**
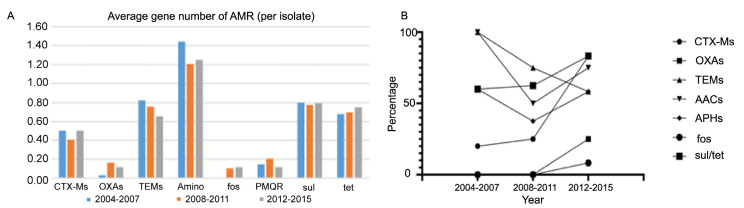
Average number of genes targeting crucial antibiotics including *bla*_CTX-M_, *bla*_TEM_, and *bla*_OXA_ in all the isolates (**A**) and in ST131 (**B**) in different time periods.

Next, we investigated multiple CARD items targeting crucial antibiotics which were frequently reported worldwide among the isolates. Specifically, we focused on beta-lactamases, including *bla*_CTX-M_, *bla*_TEM_, *bla*_OXA_, and *bla*_AmpC_; as well as *ant*, *aac*, and *aph* genes, responsible for aminoglycoside resistance; *qep, oqx*, and *qnr*, responsible for quinolone resistance (also known as plasmid-mediated quinolone resistance, PMQR genes); alongside other common antibiotic resistance genes such as *tet*, *sul*, and *fos*. By analyzing the average gene number for each CARD item in the isolates, we observed that the number of aminoglycoside resistance genes was the highest, followed by *bla*_TEM_, *sul*, and *tet*. Despite some variations across different time periods, the predominance of these AMR items remained consistent. Additionally, a slight decreasing trend with time of bla_TEM_ was identified (Figure 4A).

In our investigation of the CARD gene profile within STs, we found a significant decrease in the prevalence of *bla*_TEM_ over time across all isolates, whereas *bla*_CTX-M_, *bla*_OXA_, *fos*, *sul*, and *tet* prevalance increased in ST131 isolates (Figure 4B).

### 3.6. Plasmid-Mediated Antibiotic-Resistant Genes

**Figure 5:**
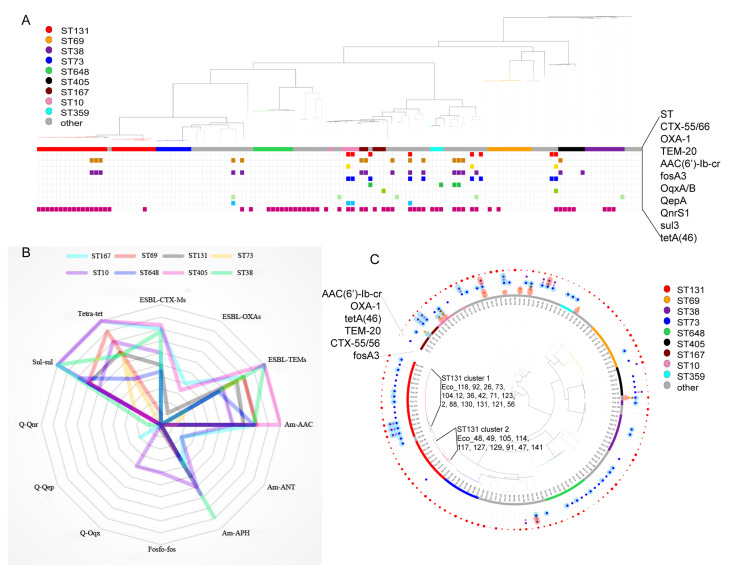
Antibiotic resistance genes on plasmid. (**A**) Comparison of AMR genes *bla*_CTX-55/56_, *bla*_OXA-1_, *bla*_TEM-20_, *aac(6′)-Ib-cr*, *fosA3*, *oqxA/B*, *qepA*, *qnrS1*, *sul3,* and *tetA(46)* in different STs. Heatmap was made based on the number of AMR genes in different STs; (**B**) Spectrum of frequently reported antibiotic resistance genes in different STs in the radar graph; (**C**) Co-occurrence of antibiotic resistance genes on plasmid and plasmid replicon types in different STs. The ring from the inner to the outer is *fosA*, *bla*_CTX-55/56_, *bla*_TEM-20_, *tetA(46)*, *bla*_OXA-1_, *aac(6′)-Ib-cr*. IncF and IncHI2 are marked in blue and red, respectively. If two or more genes were present in the same genome, the location of the genes in the genome was analyzed to verify co-occurrence.

Plasmids play an important role in antibiotic resistance. Although second-generation sequencing cannot obtain complete plasmid sequences, some antibiotic resistance genes can be characterized as plasmid-borne by blasting contigs harboring antibiotic resistance genes with known plasmid sequences. Upon further investigation, we identified certain AMR genes located on plasmid sequences, including *bla*_CTX-55/56_, *bla*_OXA-1_, *bla*_TEM-20_, *aac(6′)-Ib-cr*, *fosA3*, *oqxA/B*, *qepA*, *qnrS1*, *sul3,* and *tetA(46)* (Figure 5A). Among the 137 *E. coli* isolates, *tetA(46)* was found to be widely distributed, which is consistent with the pervasive resistance to tetracyclines. In comparison to the other STs, ST73 exhibited fewer plasmid-mediated AMR genes, as indicated in Figure 5A, which is consistent with the limited antibiotic resistance gene spectrum of ST73 depicted in the radar graph of Figure 5B. Plasmid-mediated quinolone resistance genes (*qnr*, *qep*, and *oqx*) were only present in ST167, ST359, and other non-dominant sequence types, whereas the plasmid-mediated *fos* gene was only found in ST10 and other types. All the isolates in ST131 cluster 1 in the core genome tree have *tetA (46)* or other plasmid-mediated genes such as *bla*_OXA-1_ and *aac(6′)-Ib-cr*, while, except Eco_91, none of the isolates in ST131 cluster 2 has any plasmid-mediated genes (Figure 5C).

Furthermore, we identified several co-harboring relationships between certain plasmid-related genes. It is evident that there is a correlation between the presence of *aac(6′)-Ib-cr* and *bla*_OXA-1_ (Figure 5C). Upon identifying their precise locations in the genome sequences, we found that these two genes clustered on the same contig of the same plasmid in each strain. *bla*_CTX-M-55/56_ and *fosA3* also exhibit a degree of co-occurrence. While we also determined the contig locations of other genes, we did not discover such co-existence. However, genes distributed on different contigs may still be situated on the same plasmid, possibly as a result of incomplete assembly. We further analyzed the replicon of each plasmid contig carrying these genes. Two plasmid replicon types, IncF and IncHI2, were primarily identified, as annotated in Figure 5C. It is evident that *aac(6′)-Ib-cr* and *bla*_OXA-1_ are typically carried by IncF plasmids, and sometimes by IncHI2. Most *tetA* genes were carried by IncF plasmids, whereas most *fosA3* genes were located on IncHI2.

### 3.7. Comparison of Virulence Genes Among Different STs

**Figure 6:**
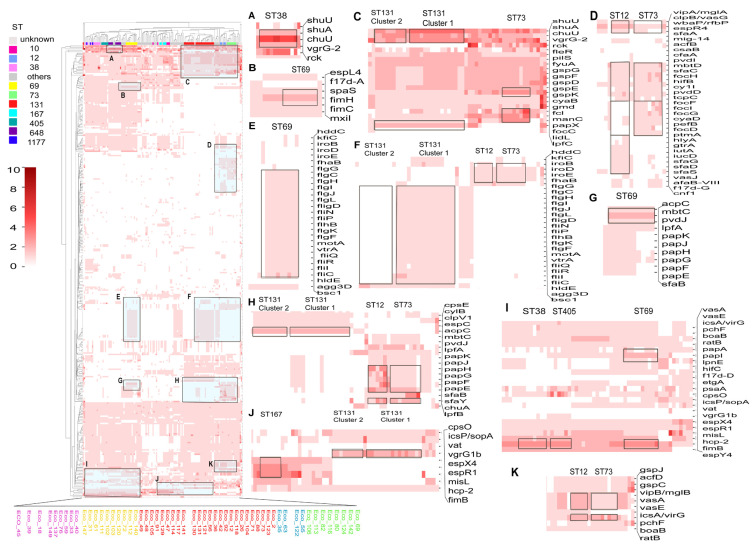
Clustering heatmap based on Virulence Factor Database (VFDB) gene content. STs are marked at the top. Intensity of box shading indicates the number of antibiotic genes related to the VFDB item. (**A**,**B**) abundant VFDB items in ST38; (**B**,**E**,**G**,**I**) abundant VFDB items in ST69; (**C**,**D**,**F**,**H**,**J**,**K**) the difference in VFDB items among ST131 cluster 1, cluster 2, ST73, and ST12.

Similarly, a heatmap was created based on VFDB gene content in each isolate for the main STs to reveal abundant virulence genes in different STs. Overall, ST73 and ST12 demonstrated a pronounced advantage in terms of virulence, possessing many abundant VFDB genes (Figure 6). ST73 and ST12 exhibited similar characteristics in terms of virulence genes, with some commonly held genes such as *pvdI/D*, *mbtD*, *sfaB/C*, *focH*, *hifB*, *cylI*, *tcpC*, *iroB/D/E*, *fhaB*, *chuA*, and *pchF*. ST73 harbored a large number of unique genes: *cyaB/D*, *papX*, *focC/F/I/G/D/H*, *pefB*, and *ptmA*. ST12 harbored a large number of *vasG/J/A/E*, *iutA*, *iucD*, *sfaD/G/S*, *papH/G/F/E*, and *vipB* (Figure 6D,F,H,K). In comparison, ST131 and ST 69 also exhibited their unique abundant virulence genes. ST131, in general, possessed numerous *lpfC*, *cpsE*, *cylB*, *clpV1*, *espC*, and *vgrG1b* genes (Figure 6C,H,J). At the same time, ST131 was grouped into two clusters as per the virulence heatmap. Interestingly, the two clusters based on VFDB are highly consistent with the core genome tree, with only two isolates (Eco_127 and Eco_73) differing from the core genome tree. Cluster 1 (Eco_2, 123, 73, 88, 71, 104, 26, 118, 12, 92, 42, 36, 56, 121, 131, 130) is richer in virulence genes than cluster 2 (Eco_141, 117, 114, 47, 129, 91, 105, 48, 49), particularly in genes such as *shuU/A*, *flgG/C/H/I/J/L/K/F*, *fliR/Q/D/N/P/A/I/C*, *flhB*, *motA*, *vtrA*, *hldE*, and *add3D* (Figure 6C,F). It is worth mentioning that, in the CARD heatmap, Eco_36, 42, 26, 104, 92, 118, and 123 have more CARD items than the other ST131 isolates, and here, these isolates also have more virulence genes than the other ST131 isolates. For the cluster 1-abundant virulence genes, *flgG/C/H/I/J/L/K/F*, *fliR/Q/D/N/P/A/I/C*, *flhB*, *motA*, *vtrA*, *hldE*, and *add3D* were also abundant in ST69 (Figure 6E). ST69 also showcased unique gene abundance, such as *fimH*, *mbtC*, *ratB* (Figure 6B,G,I); *chuU* was more pronouncedly abundant in ST38 (Figure 6A). ST167 also featured distinct genes, such as *espR4/X4/R1*, *misL* (Figure 6J).

### 3.8. Comparison of Secretion Systems and Genomic Islands Among Different STs

Additionally, analysis for secretion system-related coding genes showed that ST131, ST12, ST73, and ST1193 exhibited more type II secretion systems; furthermore, ST131, ST12, and ST1193 demonstrated more type VI secretion systems than the other STs (Figure S1). As indicated in Figure 1B, the four STs were clustered in the lower right cluster, based on PCA analysis.

**Figure 7:**
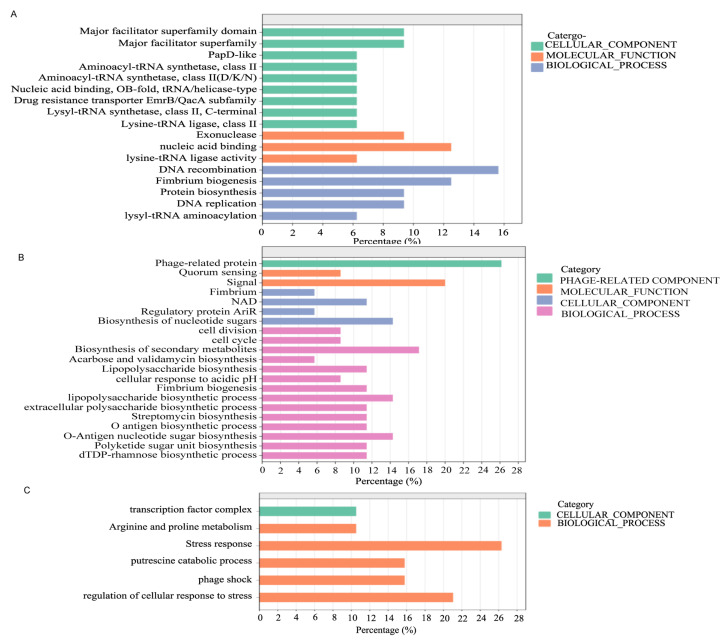
GO functional analysis of the genes in the genomic island in ST131 (**A**), ST73 (**B**), and ST167 (**C**).

Based on the distribution of gene islands in different isolates, a heatmap was generated (Figure S2), showing different STs have their own abundant genomic islands. The genes on these genomic islands were subjected to GO functional analysis (Figure 7). The functional analysis of unique genomic islands for ST131 showed that the abundant genes were primarily involved in DNA recombination, nucleic acid binding components, and the biological development of pili, which was also evident in the BRIG circle diagram comparison (Figure S3). The functional analysis of unique gene islands for ST73 primarily involved lipopolysaccharide formation, O antigen synthesis, and pilus synthesis processes, reflecting the apparent virulence advantage of ST73. The functional analysis of unique gene islands for ST69 was largely associated with external stress response, with a notable emphasis on the stress response to bacteriophage fragment insertion.

## 4. Discussion

The present study offers a compelling genomic perspective on the evolution of antibiotic resistance and virulence in *E. coli* isolates over a 12-year period within a Chinese hospital setting. Our analysis identified ST131, ST69, ST648, ST38, and ST73 as the most prevalent sequence types, corroborating the findings of other studies and highlighting the global predominance of these clones [29,30,31]. Our findings elucidated an alarming trend of increasing resistance to a broad spectrum of antibiotics, including those that are cornerstones of modern antimicrobial therapy, such as β-lactams, aminoglycosides, and quinolones. The predominance of MDR isolates, especially within the ST131 clonal group, underscores the urgency for novel therapeutic strategies. This pattern of resistance is not only confined to our study but is also reflective of a global crisis in antibiotic resistance.

### 4.1. Evolution of Antibiotic Resistance and Virulence

Bacterial antibiotic resistance and virulence have traditionally been regarded as distinct evolutionary entities. We found a strong correlation between phenotypic and genotypic resistance. Specifically, the isolates exhibited a high prevalence of antibiotic resistance genes associated with penems and fluoroquinolones, which correlated with the high rates of resistance observed in phenotypic testing: 87% for penicillins and 61% for fluoroquinolones. Furthermore, our phylogenetic analysis (Figure 1) revealed distinct clustering patterns, with multidrug-resistant (MDR) isolates and sensitive isolates forming separate groups. This separation underscores modes of transmission of antibiotic resistance in these isolates, suggesting clonal expansion of different MDR and susceptible strains rather than horizontal gene transfer of antibiotic resistance genes among different strains. As expected, when comparing the CARD heatmap data, we observed that MDR isolates harbored a significantly higher number of antibiotic resistance genes compared to sensitive strains. These findings collectively support the concordance between phenotypic and genotypic resistance traits.

Throughout the typing process, a distinct correlation was observed between each ST type and its corresponding antibiotic and virulence genes, suggesting a level of uniformity within the bacterial clusters. ST131 was found to possess a high degree of antibiotic resistance, whereas ST73 and ST12 appeared to have lower levels of resistance. This difference in antibiotic susceptibility patterns among various ST types points to the complex nature of bacterial evolution and adaptation. CARD analysis provided a partial explanation for the observed resistance phenotypes among the bacteria. ST73, both in the CARD gene heatmap analysis and the resistance gene content analysis of key antibiotics, showed a significant lack of resistance genes compared to other STs. This may underline the observed prevalence of sensitive isolates within ST73. Previous studies comparing ST131 and ST73 have consistently indicated that ST73 does not possess the same level of resistance as ST131 [32], suggesting that these two types may employ divergent strategies for survival, potentially contributing to their status as dominant isolates in multiple studies. On the other hand, ST73 and ST12, overall, showed greater prominence in virulence genes, potentially compensating for their relative lack of resistance genes. The abundance of these virulence genes may account for ST73’s emergence as a dominant isolate type. Compared to ST131, ST73’s unique virulence genes are predominantly involved in pili coding, while the abundant virulence genes in ST73 and ST12 appear more diverse and extensive. This diversification of virulence genes is a significant factor in ST73’s rise as a prevalent type. The identification of unique virulence genes within specific ST types, such as ST73 and ST12, may offer targets for the development of isolate-specific interventions.

### 4.2. Convergence of Resistance and Virulence

Importantly, evidence of intra-ST type clustering was also identified. Our further analysis demonstrated that the observed intra-ST type clustering was not merely a superficial attribute, but rather reflected significant genetic variations which have the potential to generate convergent isolates with high virulence and antibiotic resistance within the same ST. For instance, for ST131, the clustering observed in the VFDB heatmap aligns highly with the core genome tree, with cluster 1 being characterized by a higher number of virulence genes compared to cluster 2. This alignment suggests that virulence factors may be conserved across closely related isolates, providing valuable insights into the evolution and dissemination of virulence traits. Upon scrutinizing the CARD item heatmap for ST131, a more complex picture was observed. Despite the overall similarity in the clustering pattern with the core genome tree, significant discrepancies are evident, suggesting horizontal antibiotic-resistant elements among different isolates. The presence of isolates like Eco_36, 42, 26, 104, 92, 118, and 123, which exhibit both high virulence gene counts and relatively high CARD item counts, is particularly noteworthy. Further analysis showed that these isolates all have plasmid-mediated antibiotic resistance genes (Figure 5), and may represent a unique class of virulence and resistance convergent isolates. Historically, bacterial antibiotic resistance and virulence have been considered as non-overlapping populations, with only the occasional and sporadic report of a hypervirulent isolate acquiring antimicrobial resistance in *K. pneumoniae* [33]. Our study revealed a potential link between these two phenomena in *E. coli*. To our knowledge, this is the first time the potential convergence of resistance and virulence in *E. coli* has been revealed. It is thus evident that for isolates with high virulence, the acquisition of antibiotic resistance genes facilitates the transition to becoming both highly virulent and highly resistant, thereby representing a substantial threat to public health.

### 4.3. Implications of Horizontal Gene Transfer

Plasmids have been identified as pivotal vehicles for the dissemination of antibiotic resistance genes. The co-existence of certain resistance genes on the same plasmid, such as *aac(6′)-Ib-cr* and *bla*_OXA-1_, suggests a mechanism for the rapid spread of antibiotic resistance within *E. coli* populations. The prevalence of IncF and IncHI2 plasmids in our samples suggests that these replicons may be major contributors to the spread of antibiotic resistance determinants among *E. coli* isolates, and thus warrant more attention. Particularly, in this study, plasmids carrying these two genes are not only present in certain mainstream isolates of ST131, ST167, and ST405, but also in some A-group and B1-group isolates.

At the same time, we found a significant increase in *bla*_CTX-M_, *bla*_OXA_, *fos*, *sul*, and *tet* genes over time in ST131; this may be indicative of selective pressures exerted by the clinical use of corresponding antibiotics and contribute to the sustained dominance of ST131 over the course of 12 years, and could potentially indicate a lasting trend in the subsequent years. Recently, a study indicated OXA-244 is emerging in Europe, and OXA-244-producing ST131 *E. coli* were reported [34].

The analysis of genomic islands within different STs provides further insight into the genetic basis of *E. coli* pathogenicity. The unique virulence islands identified in ST73, which are abundant in genes related to lipopolysaccharide endotoxin and pili, may contribute to the increased invasiveness of this ST type. Conversely, the genomic islands of ST131, associated with DNA recombination and bacteriophage insertion, may predispose this ST type to the acquisition of foreign genes, thereby bolstering its resistance and virulence profiles, continually enhancing its competitive advantage, and maintaining its position as the most prevalent type over the 12 years.

### 4.4. Clinical and Public Health Implications

The findings of this study underscore the necessity for enhanced surveillance and early warning systems for *E. coli* antibiotic resistance and virulence. Promoting the judicious use of antibiotics and bolstering host immunity are crucial in curbing the spread of *E. coli* infections. Additionally, the development of targeted strategies for different ST types of *E. coli* could be instrumental in managing hospital-acquired infections.

There are also some limitations in this study. First, the relatively small number of isolates collected annually limited our ability to identify statistically significant trends in the prevalence of antibiotic resistance and virulence genes over time or within specific infection types. Future studies should aim to include a larger sample size to better evaluate temporal and infection-type-specific patterns. Second, the findings of this study are based on isolates collected from a single hospital in China, which may restrict the generalizability of our results to global *E. coli* populations. Future multi-center studies are needed to validate these trends. Thirdly, the samples were collected between 2004 and 2015; ongoing surveillance and future research, including up-to-date isolates to track the progression of resistance patterns, are needed.

## 5. Conclusions

This study unveiled a stark and concerning trend in the genetic evolution of antibiotic resistance and virulence in *E. coli* isolates over a 12-year period. Our study bridges a notable gap in the longitudinal research landscape pertaining to *E. coli*. We found high intra-clonal diversification and convergence of antibiotic resistance and virulence in the dominant group ST31, suggesting frequent variation in the *E. coli* population under antibiotic pressure. Our findings underscore the urgent need for continued surveillance and monitoring of antibiotic resistance patterns in bacterial populations. Future research should focus on several key areas. Longitudinal studies monitoring the evolution of antibiotic resistance and virulence in *E. coli* are essential for predicting future resistance patterns. Further investigation into the host adaptation and transmission mechanisms of different ST types of *E. coli* is also warranted. Lastly, the development of novel antibiotics and alternative therapeutics to combat the rising tide of antibiotic resistance is a critical direction for future scientific inquiry.

## Data Availability

All data included in this study are available upon request by contact with the corresponding author.

## Supplementary Materials

The following supporting information can be downloaded at: https://www.mdpi.com/article/10.3390/microorganisms13040954/s1, Figure S1: Heatmap based on secretion system-related coding genes in different STs.; Figure S2: Heatmap based on the distribution of gene islands in different STs.; Figure S3: BRIG circle diagram comparison of ST131, ST167, and ST73. The circles from inner side to outer side represent the genomes of Eco_130, 131, 121, 91, 127, 129, 1, 14, 20, 65, 90, 69, 80, 82, 106, 113, 116, 124, 142, respectively. Table S1: The antibiotic susceptibility, STs, serogroup, and phylogroup of the isolates. Table S2: Distribution of MDR and Susceptible Strains by Phylogroup. Table S3: Top STs Associated with MDR.
