# Supplementary material for: Genetic Evolution of Antibiotic Resistance and Virulence Genes in Escherichia coli Isolates from a Chinese Hospital over a 12-Year Period
Source: Microorganisms. 2025 Apr 21;13(4):954. doi: 10.3390/microorganisms13040954 (PMC12029843; doi:10.3390/microorganisms13040954)
Supplement: Supplementary file 1 [file microorganisms-13-00954-s001.zip › Legend for Figure S1 S2 Table S1.pdf]

Legend for Figure S1-3 and Table S1

Figure S1 Heatmap based on secretion system-related coding genes in different STs.

Figure S2 Heatmap based on the distribution of gene islands in different STs.

Table S1 The antibiotic susceptibility, STs, serogroup and phylogroup, etc. of the strains.
