# Supplementary figures and images for: Genetic Evolution of Antibiotic Resistance and Virulence Genes in Escherichia coli Isolates from a Chinese Hospital over a 12-Year Period
Source: Microorganisms. 2025 Apr 21;13(4):954. doi: 10.3390/microorganisms13040954 (PMC12029843; doi:10.3390/microorganisms13040954)

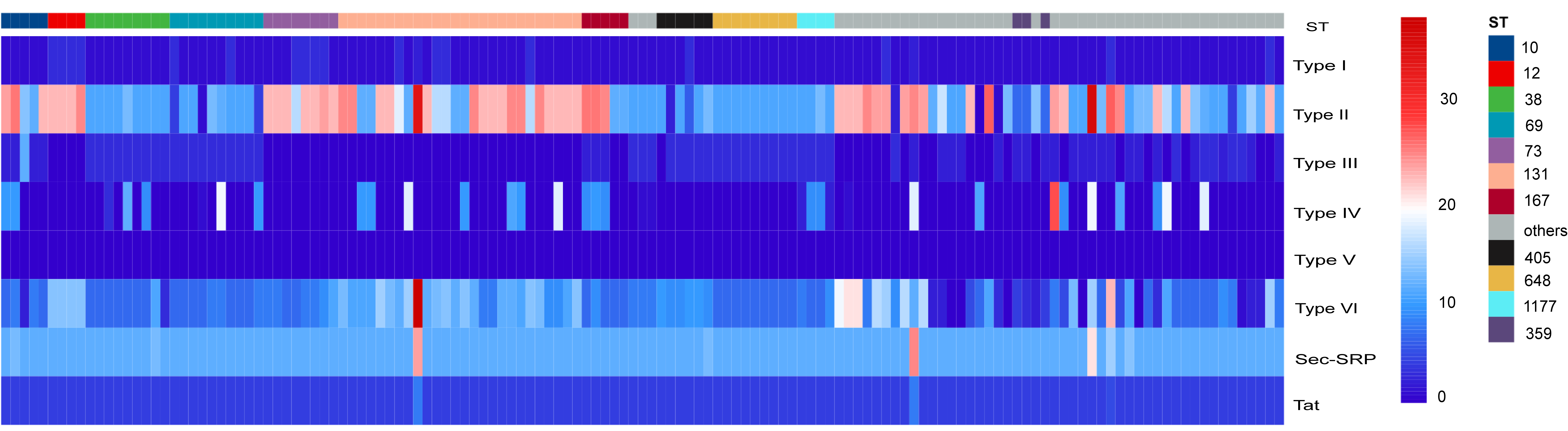

Supplement: Supplementary file 1 [file microorganisms-13-00954-s001.zip › Fig S1.tif]

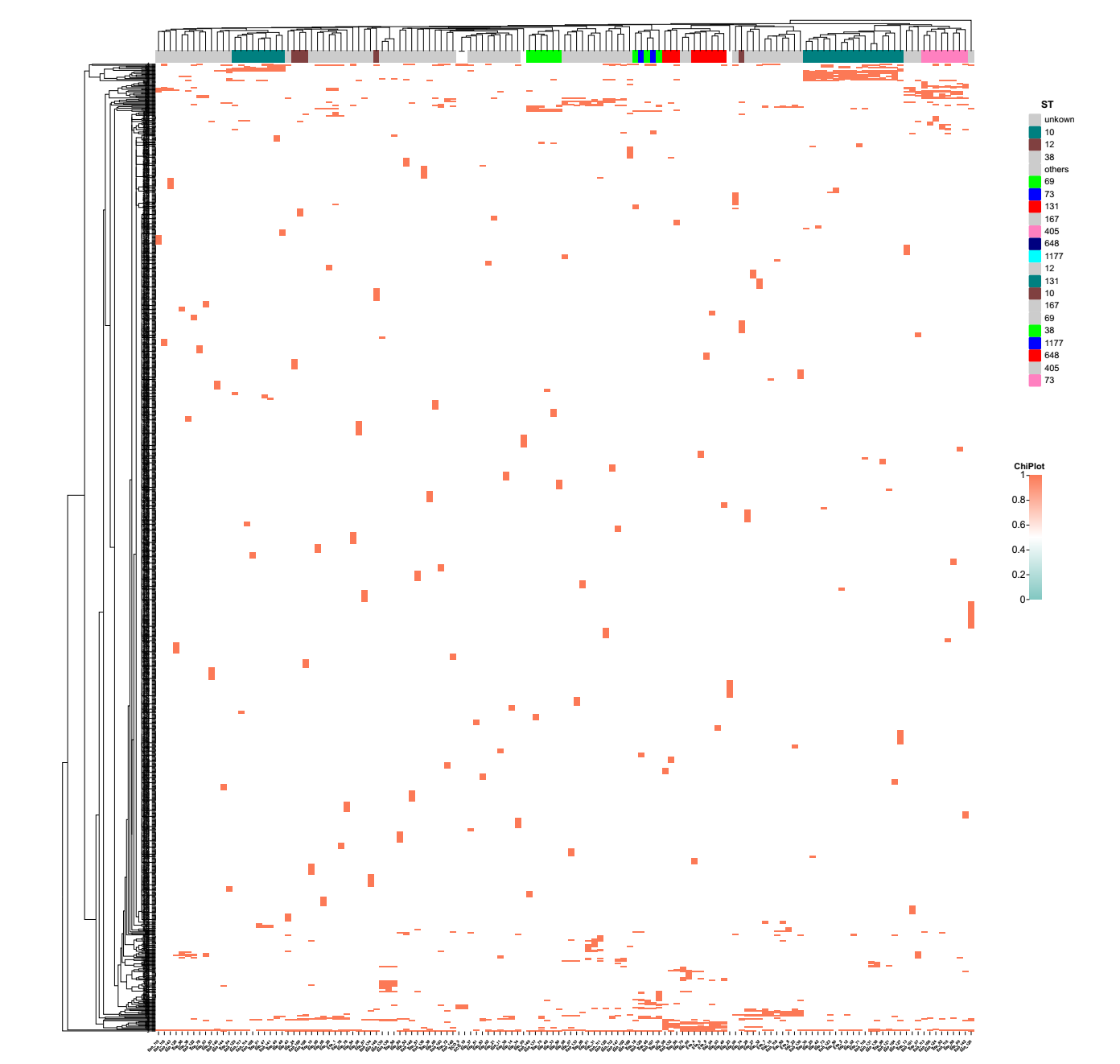

Supplement: Supplementary file 1 [file microorganisms-13-00954-s001.zip › Fig S2.tif]

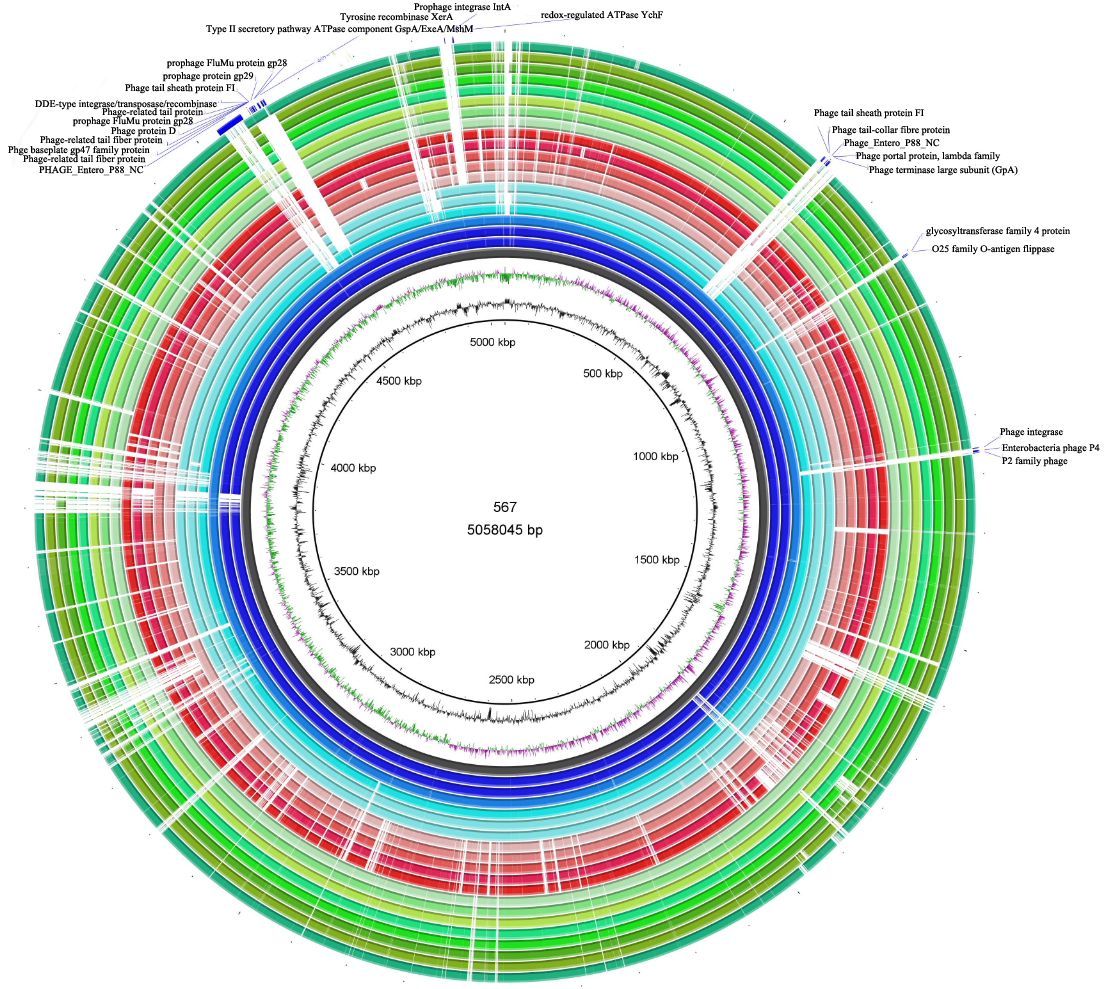

Supplement: Supplementary file 1 [file microorganisms-13-00954-s001.zip › Fig S3.tif]
